# Polymorphism of MMP-3 gene and imbalance expression of MMP-3 / TIMP-1 in articular cartilage are associated with an endemic osteochondropathy, Kashin- Beck disease

**DOI:** 10.1186/s12891-021-04952-9

**Published:** 2022-01-03

**Authors:** Bohui Shi, Xiong Guo, Aili Iv, Zengtie Zhang, Xiaowei Shi

**Affiliations:** 1 Department of Breast Surgery, The First Affiliated Hospital of Xi’an Jiaotong University, Xi’an, Shaanxi 710061 PR China; 2 School of Public Health, Xi’an Jiaotong University Health Science Center, Key Laboratory of Environment and Gene Related Diseases of Ministry of Education, Key Laboratory of Trace Elements and Endemic Diseases of Ministry of Health, Xi’an, Shaanxi 710061 PR China; 3 School of Nursing, Xi’an Jiaotong University Health Science Center, Xi’an, Shaanxi 710061 PR China; 4 Department of Paediatrics, The First Affiliated Hospital of Xi’an Jiaotong University, Xi’an, Shaanxi 710061 PR China

**Keywords:** Kashin-Beck disease, Matrix metalloproteinase-3, Tissue inhibitors of matrixmetalloproteinases-1, Single nucleotide polymorphisms, Immunohistochemistry

## Abstract

**Background:**

The etiology of Kashin-Beck disease (KBD), an endemic osteochondropathy, is largely unknown. Matrix metalloproteinase-3 (MMP-3) plays a central role in the initiation and progression of cartilage destruction, however, no study has reported on the relationship between KBD and MMP-3. The objective of this study was to explore the polymorphism of MMP-3 gene and expression of MMP-3 / TIMP-1(Tissue inhibitors of matrixmetalloproteinases-1) in the pathogenesis of KBD.

**Methods:**

Single nucleotide polymorphism (SNP) genotyping was conducted in 274 KBD cases and 248 healthy controls for eight SNPs in MMP-3 using the Sequenom MassARRAY system. Additionally, the expression of MMP-3、TIMP-1 in different layers of the articular cartilage was analyzed by immunohistochemistry for 22 KBD patients, 15 osteoarthritis (OA) patients and 21 controls.

**Results:**

The results showed that six SNPs (rs520540、rs591058、rs679620、rs602128、rs639752 and rs678815) in MMP-3 were associated with the increased risk of KBD, however, after Bonferroni correction, only the SNP rs679620 in the recessive model remained significant difference (OR = 2.31, 95%CI = 1.29–4.14, *P* = 0.0039), homozygous for “T” allele have a risk for KBD than “C” allele carriers. Moreover, the percentages of cells expressing MMP-3 in articular cartilage were significantly higher in the KBD and OA groups than in the controls (*t = 5.37 and 4.19*, *P*<0.01). While the KBD and OA groups had lower levels of TIMP-1 positive staining compared with the controls (*t = 5.23and 5.06*, *P*<0.01). And there was no significant different between KBD and OA for the levels of MMP-3 and TIMP-1 positive staining (*t = 0.05and 0.28*, *P*>0.05).

**Conclusions:**

MMP-3 is associated with the susceptibility of KBD, and the imbalance expression of MMPs / TIMPs leading to cartilage degradation may play an important role in cartilage degradation and osteoarthritis formation in OA and KBD.

## Introduction

Kashin-Beck disease (KBD) is a chronic osteochondropathy affecting the bones and joints that is endemic to certain geographical areas of Russia, North Korea and China [1]. A key pathological feature of KBD is chondrocyte necrosis in the deep zone of the growth plate of cartilage and articular cartilage [2, 3]. Clinically, the disease is mainly manifested as the pain, movement disorder and deformities of multiple joints.

The etiology of KBD remains unknown, a multifactorial model considering the interactions of the multiple environmental and genetic factors has been developed for the KBD [4]. Several susceptibility genes have been reported associated with susceptibility for KBD, such as ITPR2, GPX4 and HLA-DRB1 [5, 6]. However, the KBD risk explained by the gene loci was very limited. Matrix metalloproteases (MMPs) are considered to play an important role in the pathogenesis of osteoarthritis (OA) for they can degrade almost all extracellular matrix (ECM) of cartilage [7]. MMP-3 is the most important protease involved in cartilage degradation, and it can be activated by cytokines such as IL-1 and TNF-a. Following activation, MMP-3 can activate the other MMPs and degrade multiple proteins, including fibronectin, cartilage link protein, and collagen types IV, VII, IX, and XI [8], the activitie of MMP-3 can be balanced by tissue inhibitor of metalloproteinase-1 (TIMP-1). The balance between the activity of MMPs and that of TIMPs has been considered to affect the integrity of connective tissue, including cartilage [9]. Studies on the genetic polymorphisms of OA have found that the polymorphisms of MMP-3 promoter 5A / 6A are associated with this disease and MMP-3 is thought to be important in destructive joint change such as OA and RA [10]. KBD is a chronic secondary osteoarthropathy, however, little research has reported on the relationship between KBD and MMP-3. In this study, for the first time, MMP-3 genetic polymorphisms and protein expression of MMP-3 and TIMP-1 were studied to evaluate their effects on the risk of KBD.

## Methods

### Study populations

The samples for polymorphism of MMP-3 gene study were the same as our preliminary research [11]. In total, 274 KBD patients and 248 frequency-matched by age (53.37 ± 10.79 vs 51.71 ± 17.85, t = 1.29, *P* > 0.05) and sex (male/female, 125/149 vs 124/124, *x*^2^ = 1.01, *P* > 0.05) healthy controls were included in the current study, these unrelated individuals were from KBD-endemic areas of the Linyou and Yongshou counties of Shaanxi province, in northwest China. Radiographs of the right hand were taken for both the KBD patients and the healthy controls and read by veteran orthopedists. KBD was diagnosed according to the national diagnostic criteria of China (WS/T 207–2010). A healthy case was defined as having neither KBD nor arthritis. Participants with genetic bone and cartilage diseases, clinical symptoms or radiographic changes of other osteochondropathy were excluded. Fresh blood (5 mL) was collected from the antecubital vein of all 522 subjects while in a fasting state.

Samples for immunohistochemistry: Totally, 58 knee cartilage specimens were used in this study. Specimens of KBD and OA were collected from 22 KBD patients and 15 primary OA patients undergoing total knee replacement surgery. Normal knee cartilage specimens were collected from 21 subjects undergoing amputation caused by traffic accidents. The specimens of 22 KBD patients and 21 healthy controls were the same as our preliminary study [11], the KBD patients, consisting of ten males and twelve females with an average age of 51.00 ± 8.30 (32–66) years; The healthy control subjects, consisting of eleven males and ten females with an average age of 48.23 ± 7.65 (33–61) years; The OA patients, consisting of eight males and seven females with an average age of 51.00 ± 6.09(39–64) years. No significant differences were observed among KBD、OA and control group in age (*F* = 0.86, *P* > 0.05) and sex (*x*^2^ = 0.30, *P* > 0.05).

### SNPs selection and genotyping analysis

MMP-3 gene was reported association with several osteochondropathy diseases [7, 8]. We then searched the SNPs in dbSNP database and 1000 Genomes database (http://www.internationalgenome.org/) to obtain the genetic data of them. We selected eight SNPs of the MMP3 (see Table 1) based on the minor allele frequencies (MAF) of all the selected SNPs were > 5% in the 1000 Genomes Project (http://www.internationalgenome.org/) in Chinese population. Genomic DNA was extracted from the peripheral blood using a blood DNA extraction kit (TIANGEN, Beijing, China). Genotyping was performed using the Sequenom MassARRAY system. Primers were designed using Sequenom SNP Assay Design software version 3.0 for iPLEX reactions. The protocol and reaction conditions were in accordance with the manufacturer. Data management and analysis were conducted by Sequenom Typer 4.0 Software.

**Table 1:** The loci information of the eight SNPs in MMP-3

SNPs	Alleles ^a^	SNP location	MAF ^b^ (%)	Minor allele frequency (%)			HWE ^c^ test(*P*)
				KBD	Controls	*P*	
rs650108	A/G	Intron	0.39	41.97	39.91	0.51	1.00
rs520540	G/A	Coding exon	0.31	33.94	30.04	0.18	0.76
rs591058	C/T	N/A	0.30	33.94	30.04	0.18	0.65
rs679620	C/T	Coding exon	0.31	33.03	30.04	0.29	0.29
rs602128	G/A	Coding exon	0.31	33.94	30.04	0.18	1.00
rs639752	A/C	Intron	0.29	33.94	30.04	0.18	0.65
rs678815	C/G	Intron	0.31	33.94	30.04	0.18	0.76
rs646910	T/A	Intron	0.07	8.03	9.07	0.55	1.00

^a^ Stands for the major/minor alleles; ^b^ minor allele frequencies; ^c^ Hardy-Weinberg Equilibrium

### Immunohistochemistry

The cartilage tissues were fixed in 4% (w/v) paraformaldehyde following collection, then washed in phosphate-buffered saline (PBS), decalcified, embedded in paraffin, and cut into 5–8 μm-thick slices for immunohistochemistry [11]. Immunochemical identification was performed using the streptavidin-peroxidase (SP) method and the operations refer to our previous research [11]: Briefly, after deparaffinization, endogenous peroxidase was blocked with 3% H_2_O_2_ for 15 min, subsequently the slides were washed with PBS, and predigested using a digestive complex. After blocking using 10% normal goat serum, the sections were incubated with a primary antibody recognizing MMP-3、TIMP-1 at 1:100 dilution (polyclonal rabbit anti-MMP-3、TIMP-1, Bioss Co, Beijing, China) or with PBS (serving as a negative control) at 4 °C overnight. Next, the sections were incubated with 1:200 biotinylated goat anti-rabbit IgG (ZSGB-Bio Co, Beijing,China) at 37 °C for 20 min, followed by incubation with horseradish peroxidase-labeled streptavidin solution at 37 °C for 15 min. Color development was continued for 5 min using diaminobenzidine followed by rinsing with distilled water. Counterstaining was performed with hematoxylin.

The percentage of positive cells was obtained by counting within 10 high-magnification- power fields (40×) in six consecutive tissue sections [11].

### Statistical analyses

The Hardy-Weinberg equilibrium (HWE) of each SNP was tested to compare the expected frequencies of genotypes in controls, SNPs with *P* > 0.05 were considered to be in HWE [12]. Differences in genotypes and allele frequencies between the KBD cases and the controls were determined using CLUMP22 software. Unconditional logistic regression analysis adjusted for age and gender was used to estimate the strength of the association through calculation of odds ratios (ORs) with their 95% confidence intervals (95%CIs). Dominant、recessive and log-additive genetic models were also evaluated and expressed as ORs with 95%CI [11]. To account for multiple testing, Bonferroni’s correction was applied. Significant associations were defined at *p* value< 0.05/8 = 0.0063 [12]. Haplotypes and haplotype frequencies were calculated using Haploview software (version 4.2). Haplotypes with frequency less than 1% were combined and the haplotype with *p* value< 0.05 was considered statistically significant.

## Results

### Association of polymorphisms of MMP-3 with KBD susceptibility

**Fig. 1:**
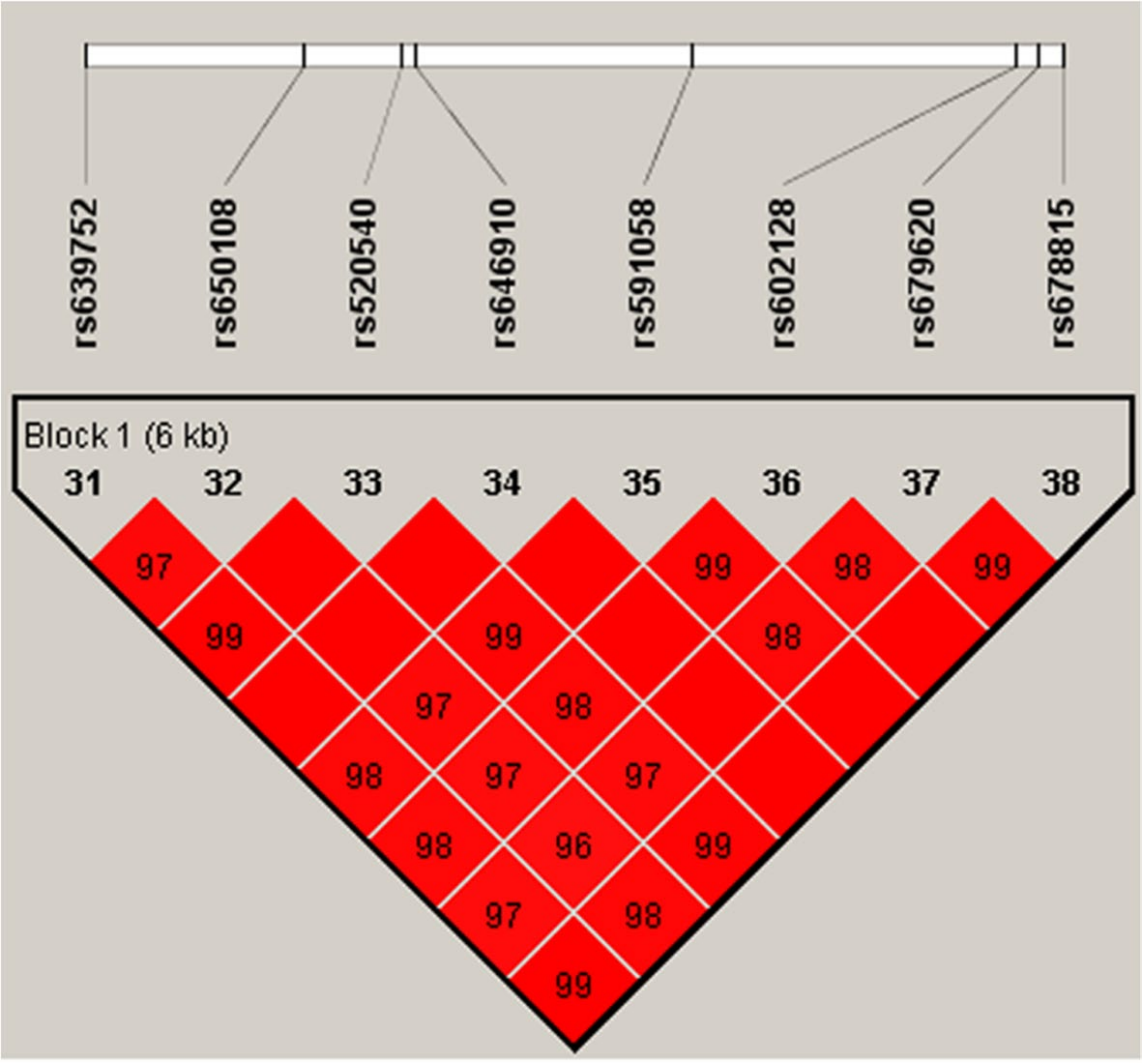
The haplotype blocks of MMP-3. The numbers indicate the extent of linkage disequilibrium based on D′ value between 2 SNPs calculated with Haploview 4.2

Table 1 showed the information of the eight SNPs, all tested SNPs were in HWE in control group (*x*^2^ = 0.02–2.41, *df* = 2, *P* = 0.29–1.00). When the allele frequencies were compared between the KBD cases and the controls, no significant association was detected in the eight SNPs (Table 1). While the distribution frequencies of genotypes in rs591058、rs679620 and rs639752 were significantly different between the two groups (Table 2). Dominant、recessive and log-additive models were applied to analyze the association between the polymorphisms and KBD, the SNPs rs520540、rs591058、rs679620、rs602128、rs639752 and rs678815 showed association with KBD in the recessive model (*P* = 0.003–0.03). However, after Bonferroni correction, only the SNP rs679620 in the recessive model remained significant association (OR = 2.31, 95% CI = 1.29–4.14, *P* = 0.0039) (Table 2), homozygous for “T” allele have a higher risk for KBD than “C” allele carriers. While the power was calculated, the results showed that our study had73.8% power to detect a nominal significant finding (alpha = 0.05) in the KBD cohort for rs679620. While the eight SNPs were analyzed for haplotypes, the results did not found any significantly (*p* > 0.05), the haplotype blocks of MMP-3 showed in Fig. 1.

**Table 2:** Analysis of association of the eight SNPs gene polymorphism with the risk of KBD

SNPs	Genotype (%) ^a^			Dominant	Recessive	Log-additive
	KBD (*N* = 274)	Controls (*N* = 248)	*P*	OR (95% CI)	OR (95% CI)	OR (95% CI)
rs650108	36.9/43.1/20.1	35.9/48.0/16.1	0.39	0.97 (0.68–1.34)	1.29 (0.82–2.01)	1.06 (0.84–1.35)
rs520540	48.2/36.9/15.0	48.0/43.1/8.9	0.07	1.00 (0.71–1.41)	1.91 (1.09–3.33) ^b^	1.15 (0.89–1.48)
rs591058	48.2/36.9/15.0	48.0/44.0/8.1	0.03 ^b^	1.02 (0.72–1.43)	1.98 (1.14–3.45) ^b^	1.17 (0.91–1.51)
rs679620	447.4/37.1/15.4	48.0/45.2/6.9	0.02 ^b^	1.00 (0.71–1.42)	2.31 (1.29–4.14) ^c^	1.16 (0.90–1.50)
rs602128	48.2/36.9/15.0	49.2/41.9/8.9	0.08	1.04 (0.74–1.47)	1.81 (1.04–3.13) ^b^	1.16 (0.90–1.50)
rs639752	48.2/36.9/15.0	48.0/45.2/6.9	0.007 ^b^	1.01 (0.71–1.42)	1.97 (1.13–3.44) ^b^	1.16 (0.91–1.54)
rs678815	48.2/36.9/15.0	48.0/43.1/8.9	0.07	1.02 (0.72–1.44)	1.91 (1.09–3.33) ^b^	1.16 (0.90–1.49)
rs646910	82.8/16.1/1.1	82.3/16.9/0.8	0.91	0.86 (0.55–1.36)	0.45 (0.04–4.99)	0.85 (0.55–1.31)

^a^Homozygote of the major allele/ heterozygote /homozygote of the minor allele

^b^The significance did not remain after correction for multiple testing

^c^The significance remained after correction for multiple testing

### Expression of MMP-3、TIMP-1 in articular cartilage of three groups

**Fig. 2:**
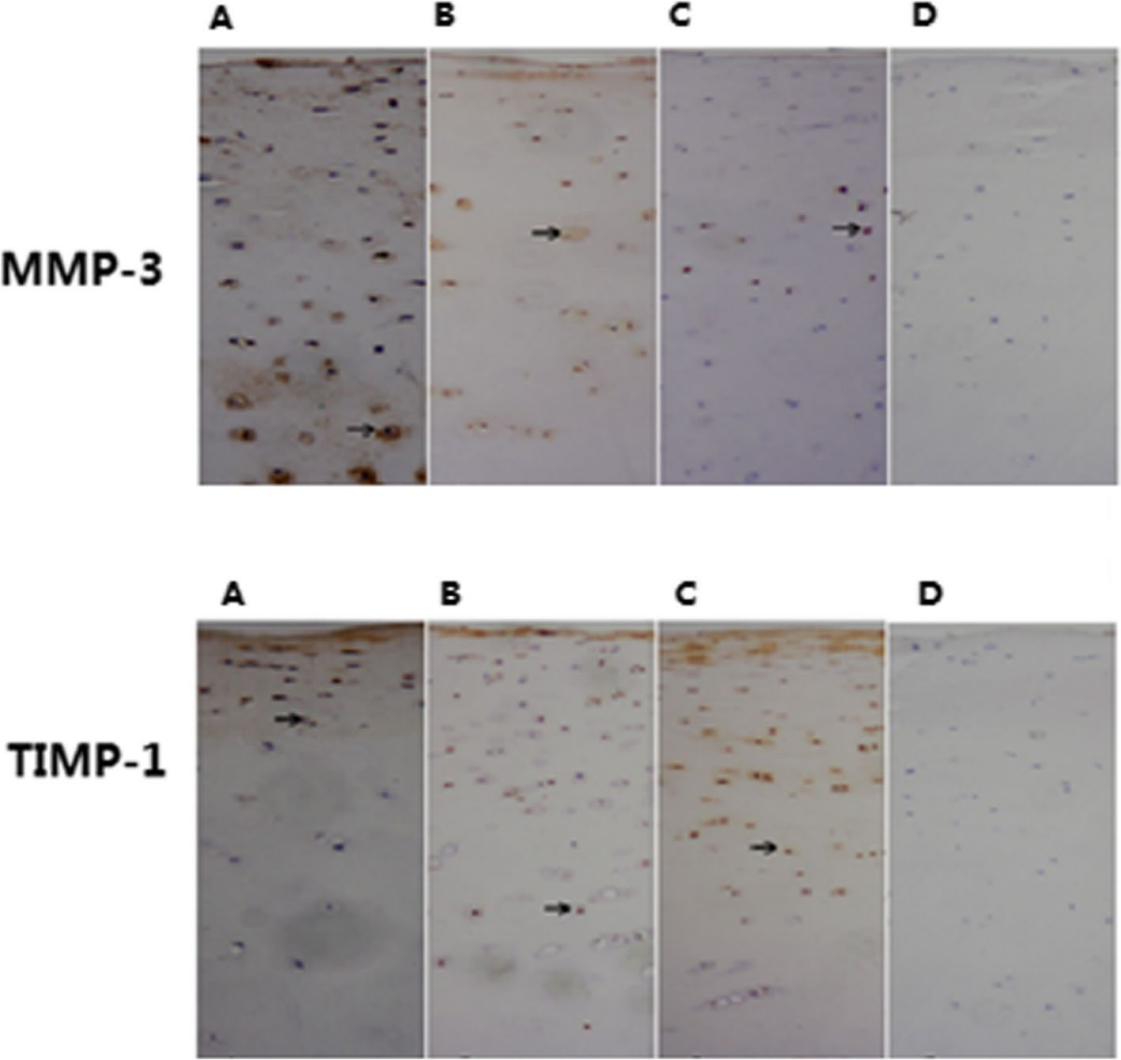
The immunohistochemical expression of MMP-3 and TIMP-1 in different layers of cartilage (Immunohistochemistry using the SP method, original magnification: ×100). **A** The cartilage of a KBD, **B** The cartilage of a OA; **C** The cartilage of a healthy control, **D** A negative control with PBS was used instead of the MMP-3 or TIMP-1primary antibody

Microscope observation revealed that MMP-3 and TIMP-1 staining was distributed throughout all zones in the articular cartilages of cases of the three groups. Starting from the uppermost layer, the percentage of chondrocytes showing MMP-3 and TIMP-1staining decreased in each successive layer of cells (Fig. 2). A comparison of the positive rate of MMP-3 stained cells in articular cartilage among the three groups showed that in each layer of the articular cartilage, the KBD cases showed a higher level of MMP-3 positive staining while a lower level of TIMP-1 positive staining than those in controls(*t* = 2.81–5.37, all *P*<0.05). Except for MMP-3 in the upper layer, the OA cases also showed a higher level of MMP-3 positive staining and a lower level of TIMP-1 positive staining compared with the controls(*t* = 2.12–5.24, all *P*<0.05). While there was no significant different between KBD and OA for the levels of MMP-3 and TIMP-1 positive staining in each layer (*t* = 0.12–1.01, all *P*>0.05) (Table 3).

**Table 3:** Immunohistochemical expression of MMP-3、TIMP-1 in different layers of the articular cartilage of the three groups

Item	Groups	n	Positive rate (%)			
			Upper layer	Middle layer	Deeper layer	Total layers
MMP-3	Control	21	46.50 ± 15.82	34.19 ± 16.00	15.48 ± 8.54	32.06 ± 6.74
	KBD	22	63.65 ± 19.78^*^	59.83 ± 20.54^*^	3.05 ± 20.92^*^	51.34 ± 15.07^*^
	OA	15	56.84 ± 20.02	57.81 ± 25.58^*^	38.68 ± 31.02^*^	51.09 ± 14.93^*^
TIMP-1	Control	21	45.78 ± 21.27	32.91 ± 16.87	16.55 ± 11.76	31.75 ± 9.87
	KBD	22	24.89 ± 22.11^*^	20.22 ± 12.53^*^	7.06 ± 5.98^*^	17.42 ± 8.09^*^
	OA	15	20.38 ± 13.75^*^	22.21 ± 11.69^*^	7.44 ± 13.77^*^	16.67 ± 7.88^*^

* *P* < 0.05 compared with the control group (*t* = 2.12–5.37, *P*<0.05)

## Discussion

KBD is an endemic OA, MMPs are considered to play an important role in the pathogenesis of osteochondropathy because they can degrade almost all ECM of cartilage. MMP-3 can directly degrade proteoglycan in the ECM and when MMP enzymes are activated, MMP-3 is activated first, then it activates the zymogens of other MMPs, which leads to degradation of ECM components [13]. The role of MMP-3 in the occurrence and development of KBD has few been investigated.

In this study, we have found that rs679620 in MMP-3 is associated with the increased risk of KBD in the recessive model in the studied populations, homozygous for “T” allele has a higher risk for KBD than “C” allele carriers. It is well documented that most diseases are associated with the individual’s genetic make-up, subtle differences in the individual genetic make-up can cause the people to respond differently to the same environmental exposure. Previous studies have indicated correlation between MMP-3 polymorphisms and different diseases including OA and RA [14–17]. Guo W et al. [14] have found that four SNPs (rs639752, rs520540, rs602128, and rs679620) in MMP-3 are associated with the increased risk of OA in Chinese men**.** Locus rs679620 is a non-synonymous SNP, the polymorphism of rs679620 may affect bone remodeling, tendinopathy, wound healing, as well as inflammatory responses and suggested that the area nearby SNP rs679620 should be the focus of additional study [18–21]. However there are limited studies for the function of rs679620 in osteoarthropathy, the mechanisms of how the MMP-3 gene contributes to KBD are needed to be clarified, further studies should be expanded including the loci nearby rs679620.

There is a balance between catabolism and anabolism in the ECM of articular cartilage which can be disturbed by certain conditions, such as inflamed synovium and pannus tissue resulting in cartilage damage [6, 22]. The present results indicate that the imbalance expression of MMP-3/ TIMP-1 in articular cartilage should play a role in the pathogenesis of KBD. MMP-3 acts as an activator of other MMPs which can degrade various ranges of component of ECM [23]. The up-regulation of MMP-3 have destructive efficacy on extracellular matrix, the activity of MMP-3 can be regulated by TIMP-1, studies showed that an excess activity of MMPs over TIMPs will result in pathologic cartilage destruction [24], our results hint the excess activity of MMP-3 over TIMP-1 plays an role in the process of KBD. MMP-3 plays an important role in the modulation of chemokines, this often initiates a cascade of degradative events through several pro-inflammatory cytokines, including IL-1 and TNF-a [25]. And studies have reported that compared with controls, levels of IL-1β and INF-α expression in synovium、serum and synovial fluid are significantly higher in patients with KBD [2, 26]. The results of this study suggest that the imbalance of MMP-3/TIMP-1 induced by IL-1β, INF-α and other causes may result in an unrecoverable degradation of the cartilage matrix, and accelerate catabolism of the ECM, thereby severely impairing ECM functionality and causing pain and disability [27] in KBD patients.

In this study, the expressions of MMP-3、TIMP-1 in articular cartilage of OA were also included, our results consistent with previous report of patients with OA showing upregulate MMP expression and downregulate TMMP expression in articular chondrocytes [25]. And in this study, we did not find significant different between KBD and OA for the level of MMP-3 and TIMP-3 positive staining in each layer. KBD is a secondary chronic deforming osteoarthritis, microscopically, the degenerative changes in the KBD cartilage are characterized by the chondronecroses in the multiple foci of the deep zone of the cartilage, the progressive chondronecrosis from the deep layers to the surface layers in articular cartilage is different from OA [28], but progressive deterioration of cartilaginous tissue occurs in both diseases. MMP-3、TIMP-1 may play the similar functions in the pathogenesis of OA and KBD,the imbalance of MMPs / TIMPs leading to cartilage degradation may play an important role in cartilage degradation and osteoarthritis formation in OA and KBD .

In conclusion, we found that the rs679620 in MMP-3 was associated with the susceptibility of KBD, homozygous for “T” allele has a higher risk for KBD than “C” allele carriers. Imbalance expression of MMP-3/ TIMP-1 in the ECM accelerates ECM catabolism, leading to matrix degradation, cartilage degeneration and subsequent pathogenesis and progression of KBD. In addition, due to the relatively small sample size, our findings are considered preliminary and need to be validated in further studies using larger sample sizes of well-defined populations.

## Data Availability

The datasets used and analysed during the current study are available from the corresponding author on reasonable request.

## Abbreviations

KBD: Kashin-Beck disease
MMP-3: Matrix metalloproteinase-3
TIMP-1: Tissue inhibitors of matrixmetalloproteinases-1
SNP: Single nucleotide polymorphism
ECM: Extracellular matrix
TNF-a: Tumor necrosis factor alpha
MAF: Minor allele frequencies
PBS: Phosphate-buffered saline
HE: Hematoxylin and eosin
SP: Streptavidin-peroxidase
HWE: Hardy-Weinberg equilibrium

## References

[1] Duan C, Guo X, Zhang XD, Yu HJ, Yan H, Gao Y (2010). Comparative analysis of gene expression profiles between primary knee osteoarthritis and an osteoarthritis endemic to Northwestern China, Kashin-Beck disease. Arthritis Rheum.

[2] Cao J, Li S, Shi Z, Yue Y, Sun J, Chen J (2008). Articular cartilage metabolism in patients with Kashine-Beck disease: an endemic osteoarthropathy in China. Osteoarthr Cartil.

[3] Mathieu F, Begaux F, Lan ZY, Suetens C, Hinsenkamp M (1997). Clinical manifestations of Kashin-Beck disease in Nyemo Valley, Tibet. Int Orthop.

[4] Liu H, Yu F, Shao W, Ding D, Yu Z, Chen F (2018). Associations between selenium content in hair and Kashin-Beck disease/ Keshan disease in children in northwestern China: a prospective cohort study. Biol Trace Elem Res.

[5] Shi Y, Lu F, Liu X, Wang Y, Huang L, Liu X (2011). Genetic variants in the HLA-DRB1 gene are associated with Kashin-Beck disease in the Tibetan population. Arthritis Rheum.

[6] Zhang F, Wen Y, Guo X, Zhang Y, Wang X, Yang T (2015). Genome-wide association study identifies ITPR2 as a suscep-tibility gene for Kashin-Beck disease in Han Chinese. Arthritis Rheumatol.

[7] Murphy G, Knäuper V, Atkinson S, Butler G, English W, Hutton M (2002). Matrix metalloproteinases in arthritic disease. Arthritis Res.

[8] Mehana E-SE, Khafaga AF, El-Blehi SS (2019). The role of matrix Metalloproteinases in osteoarthritis pathogenesis: an updated review. Life Sci.

[9] Kageyama Y, Miyamoto S, Ozeki T, Hiyohsi M, Suzuki M, Nagano A (2000). Levels of rheumatoid factor isotypes, metalloproteinase-3 and tissue inhibitor of metalloproteinase-1 in synovial fluid fromvarious arthritides. Clin Rheumatol.

[10] Abd-Allah SH, Shalaby SM, Pasha HF, El-Shal AS, Abou El-Saoud AM (2012). Variation of matrix metalloproteinase 1 and 3 haplotypes and their serum levels in patients with rheumatoid arthritis and osteoarthritis. Genet Test Mol Biomarkers.

[11] Shi XW, Lv AL, Ma J, Zhang F, Wen Y, Zhang ZT (2016). Investigation of MMP-1 genetic polymorphisms and protein expression and their effects on the risk of Kashin-Beck disease in the northwest Chinese Han population. J Orthop Surg Res.

[12] Shi XW, Zhang F, Lv AL, Wen Y, Guo X (2015). COL9A1 Genepolymorphism is associated with Kashin-Beck disease in a northwest Chinese Hanpopulation. PLoS One.

[13] Sun S, Bay-Jensen AC, Karsdal MA, Siebuhr AS, Zheng QL, Maksymowych WP (2014). The active form of MMP-3 is a marker of synovial inflammation and cartilage turnover in inflammatory joint diseases. BMC Musculoskelet.

[14] Guo W, Xu P, Jin T, Wang J, Fan D, Hao Z (2017). MMP-3 gene polymorphisms are associated with increased risk of osteoarthritis in Chinese men. Oncotarget..

[15] Ma MJ, Liu HC, Qu XQ, Wang JL (2015). Matrix metalloproteinase-3 gene polymorphism and its mRNA expression in rheumatoid arthritis. Genet Mol Res.

[16] Urata Y, Uesato R, Tanaka D, Nakamura Y, Motomura S (2012). Treating to target matrix metalloproteinase 3 normalisation together with disease activity score below 2.6 yields better effects than each alone in rheumatoid arthritis patients: T-4 study. Ann Rheum Dis.

[17] Zhu Y, Li S, Huang Z, Xing W, Li F, Da Y (2019). Association study between matrix metalloproteinase-3 gene (MMP3) polymorphisms and ankylosing spondylitis susceptibility. Mol Genet Genomic Med.

[18] Taylor J, Sun YV, Chu J, Mosley TH, Kardia SL (2008). Interactions between metallopeptidase 3 polymorphism rs679620 and BMI in predicting blood pressure in African-American women with hypertension. J Hypertens.

[19] Raleigh SM, van der Merwe L, Ribbans WJ, Smith RK, Schwellnus MP, Collins M (2009). Variants within the MMP3 gene are associated with Achilles tendinopathy: possible interaction with the COL5A1 gene. Br J Sports Med.

[20] Menezes-Silva R, Khaliq S, Deeley K, Letra A, Vieira AR (2012). Genetic susceptibility to periapical disease: conditional contribution of MMP2 and MMP3 genes to the development of periapical lesions and healing response. J Endod.

[21] Letra A, Silva RM, Rylands RJ, Silveira EM, de Souza AP, Wendell SK (2012). MMP3 and TIMP1 variants contribute to chronic periodontitis and may be implicated in disease progression. J Clin Periodontol.

[22] Zhang A, Cao JL, Yang B, Chen JH, Zhang ZT, Li SY (2010). Effects of moniliformin and selenium on human articular cartilage metabolism and their potential relationships to the pathogenesis of Kashin-Beck disease. J Zhejiang Univ-Sci B.

[23] Li J, Zhou XD, Yang KH, Fan TD, Chen WP, Jiang LF (2014). Hinokitiol reduces matrix metalloproteinase expression by inhibiting Wnt/beta- catenin signaling in vitro and in vivo. Int Immunopharmacol.

[24] Zhong Y, Huang Y, Santoso MB, Wu LD (2015). Sclareol exerts anti-osteoarthritic activities in interleukin-1β-induced rabbit chondrocytes and a rabbit osteoarthritis model. Int J Clin Exp Pathol.

[25] Wang GW, Wang MQ, Wang XJ, Yu SB, Liu XD, Jiao K (2010). Changes in the expression of MMP-3, MMP-9, TIMP-1 and aggrecan in the condylar cartilage of rats induced by experimentally created disordered occlusion. Arch Oral Biol.

[26] Sun ZM, Ling M, Liu M, Zhang YG (2009). Expression of interleukin-1β and tumor necrosis factor-α in the synovium and synovial fluid of patients with Kashin-Beck disease and osteoarthritis. J South Med Univ.

[27] Cunnane G, Fitzgerald O, Beeton C, Cawston TE, Bresnihan B (2001). Early joint erosions and serum levels of matrix metalloproteinase 1, matrix metalloproteinase 3, and tissue inhibitor of metalloproteinases-1 in rheumatoid arthritis. Arthritis Rheum.

[28] Shi XW, Guo X, Ren FL, Li J, Wu XM (2010). The effect of short tandem repeat loci and low selenium level on endemic osteoarthritis in China. J Bone Joint Surg Am.

