# Correction to: Polymorphism of MMP-3 gene and imbalance expression of MMP-3 / TIMP-1 in articular cartilage are associated with an endemic osteochondropathy, Kashin- Beck disease

**DOI:** 10.1186/s12891-022-05085-3

**Published:** 2022-02-15

**Authors:** Bohui Shi, Xiong Guo, Aili Iv, Zengtie Zhang, Xiaowei Shi

**Affiliations:** 1 Department of Breast Surgery, The First Affiliated Hospital of Xi’an Jiaotong University, Xi’an, Shaanxi 710061 PR China; 2 School of Public Health, Xi’an Jiaotong University Health Science Center, Key Laboratory of Environment and Gene Related Diseases of Ministry of Education, Key Laboratory of Trace Elements and Endemic Diseases of Ministry of Health, Xi’an, Shaanxi 710061 PR China; 3 School of Nursing, Xi’an Jiaotong University Health Science Center, Xi’an, Shaanxi 710061 PR China; 4 Department of Paediatrics, The First Affiliated Hospital of Xi’an Jiaotong University, Xi’an, Shaanxi 710061 PR China

**Correction to: BMC Musculoskelet Disord 23, 3 (2022)**

**https://doi.org/10.1186/s12891-021-04952-9**

Following the publication of the original article [1] the authors requested to amend the first affiliation from *Department of Surgical Oncology, The First Affiliated Hospital of Xi’an Jiaotong University, Xi’an, Shaanxi 710061, PR China* to *Department of Breast Surgery*, *The First Affiliated Hospital of Xi’an Jiaotong University, Xi’an, Shaanxi 710061, PR China.*

The original article [1] has been updated.
